# Chaperone‐mediated autophagy degrades Keap1 and promotes Nrf2‐mediated antioxidative response

**DOI:** 10.1111/acel.13616

**Published:** 2022-05-10

**Authors:** Lin Zhu, Shulei He, Lu Huang, Dongni Ren, Tiejian Nie, Kai Tao, Li Xia, Fangfang Lu, Zixu Mao, Qian Yang

**Affiliations:** ^1^ 12644 Department of Experimental Surgery Tangdu Hospital Fourth Military Medical University Xi’an China; ^2^ 12644 Department of Neurosurgery and Institute for Functional Brain Disorders Tangdu Hospital Fourth Military Medical University Xi’an China; ^3^ Departments of Pharmacology and Chemical Biology Emory University School of Medicine Atlanta Georgia USA

**Keywords:** 6‐OHDA, CMA, Keap1‐Nrf2 pathway, oxidative stress

## Abstract

Accumulation of oxidative stress is highly intertwined with aging process and contributes to aging‐related diseases, such as neurodegenerative diseases. Deciphering the molecular machinery that regulates oxidative stress is fundamental to further uncovering the pathogenesis of these diseases. Chaperone‐mediated autophagy (CMA), a highly selective lysosome‐dependent degradation process, has been proven to be an important maintainer of cellular homeostasis through multiple mechanisms, one of which is the attenuation of oxidative stress. However, the specific mechanisms underlying this antioxidative action of CMA are not fully understood. In this study, we found that CMA directly degrades Kelch‐like ECH‐associated protein 1 (Keap1), an adaptor of E3 ligase complex that promotes the degradation of nuclear factor erythroid 2‐related factor 2 (Nrf2), which is a master transcriptional regulator in antioxidative response. Activated CMA induced by prolonged oxidative stress led to an increase in Nrf2 level by effectively degrading Keap1, contributing to Nrf2 nuclear translocation and the expression of multiple downstream antioxidative genes. Meanwhile, together with previous study showing that Nrf2 can also transcriptionally regulate LAMP2A, the rate‐limiting factor of CMA process, we reveal a feed‐forward loop between CMA and Nrf2. Our study identifies CMA as a previously unrecognized regulator of Keap1‐Nrf2 pathway and reinforces the antioxidative role of CMA.

## Graphical abstract

Our findings show that CMA is activated under oxidative condition, resulting in the degradation of Keap1 and activation of Nrf2. This protects cells against oxidative stress. Moreover, Nrf2 increases the transcription of LAMP2A gene, which in turn further activates CMA. We provide a novel mechanism by which CMA‐Nrf2 forms a positive feedback loop to augment antioxidative response and protect cells from oxidative stress.

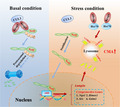

## 1 INTRODUCTION

The neuronal dysfunction or death caused by the imbalance of redox homeostasis is one of the important pathogenesis of neurodegenerative diseases, which get worse with aging (Zhang et al., 2015). The imbalance of neuronal redox homeostasis is manifested by the increase of intracellular reactive oxygen species (ROS) levels. Although the specific mechanism is not entirely clear, it is closely related to mitochondrial dysfunction, abnormal protein accumulation, lipid metabolism disorder, etc. (Liu et al., 2015; Yan et al., 2013). Therefore, maintaining redox homeostasis of neurons is critical for preventing oxidative stress‐induced neurons damage and thus the development of neurodegenerative disease with aging.

Chaperone‐mediated autophagy (CMA) is a selective protein degradation pathway through lysosomes. In CMA, substrate proteins carrying special CMA‐targeting motif (KFERQ‐like motif) are bound with Hsc70 (heat shock cognate protein 71), transferred from cytosol to the lysosomal CMA receptor LAMP2A (lysosome‐associated membrane protein type 2A), and eventually translocated into lysosome lumen for degradation (Cuervo & Wong, 2014). Emerging research has linked CMA with redox homeostasis of neurons. And the activity of CMA declines with age, due to the reduction of the protein level of LAMP2A (the rating‐limited factor of CMA), which induces or aggravates dysregulation of neuronal redox homeostasis (Cuervo & Dice, 2000; Cuervo et al., 2004). Generally, CMA is activated in response to moderate oxidative stress (Kiffin et al., 2004) and protects neurons against oxidative stress by directly degrading oxidatively damaged and nonfunctional proteins to prevent the formation of toxic and insoluble protein aggregates (Yang et al., 2009). Furthermore, we have previously demonstrated that CMA is involved in maintaining mitochondrial function and alleviating ROS production by modulating PARK7 degradation (Wang et al., 2016). However, whether CMA regulates directly the cellular response for oxidative stress remains unclear.

Nrf2 (nuclear factor erythroid 2‐related factor 2) is a master antioxidative transcription factor, which protects cell and organism against oxidative and electrophilic stresses of both exogenous and endogenous origins through binding enhancer sequences termed “antioxidant response elements” (AREs) and regulating a battery of antioxidative genes, such as NADPH quinone oxidoreductase 1 (NQO1), heme oxygenase‐1 (HMOX1), and glutathione S‐transferase M1 (GSTM1; Yamamoto et al., 2018). Nrf2 is primarily regulated by Keap1 (Kelch‐like ECH‐associated protein 1)‐Cul3 (Cullin3) E3 ligase complex (Villeneuve et al., 2010). Under basal condition, Keap1 serves as a substrate scaffold for Cul3‐containing E3 ubiquitin ligase, which induces continually ubiquitination and degradation of Nrf2 by 26S proteasome. Under oxidative stress, the reactive cysteine residues of Keap1 are directly modified, which reduces the ubiquitin E3 ligase activity of the Keap1‐Cul3 complex and results in Nrf2 stabilization and nuclear translocation for antioxidant response (Zhang et al., 2004). Besides the modification of Keap1’s cysteine residues, other mechanisms are involved in activating Nrf2. For instance, p62 (Komatsu et al., 2010), iASPP (Ge et al., 2017), and Nestin (Wang, Lu, et al., 2019) can bind to Keap1 and interfere with the interaction between Nrf2 and Keap1, which results in subsequent activation of Nrf2 signaling. Keap1 itself has been shown to be degraded by p62‐dependent macroautophagy providing a potential link between autophagy and Nrf2 (Taguchi et al., 2012).

In the present study, we demonstrate that CMA plays a critical role in Nrf2‐mediated antioxidant response in SN4741 cells (a mouse embryonic substantia nigra‐derived cell line). Our findings show that Keap1 is a direct substrate of CMA. CMA increases the level of Nrf2 by preventing it from Keap1‐mediated degradation, which leads to subsequent upregulation of antioxidant genes. CMA is activated under oxidative condition, resulting in the degradation of Keap1 and activation of Nrf2. This protects cells against oxidative stress. Moreover, Nrf2 increases the transcription of LAMP2A gene, which in turn further activates CMA. Taken together, our study provides a novel mechanism by which CMA‐Nrf2 forms a positive feedback loop to augment antioxidative response and protect cells from oxidative stress.

## 2 RESULTS

### 2.1 CMA increases Nrf2 protein level and transcriptional activity

**FIGURE 1:**
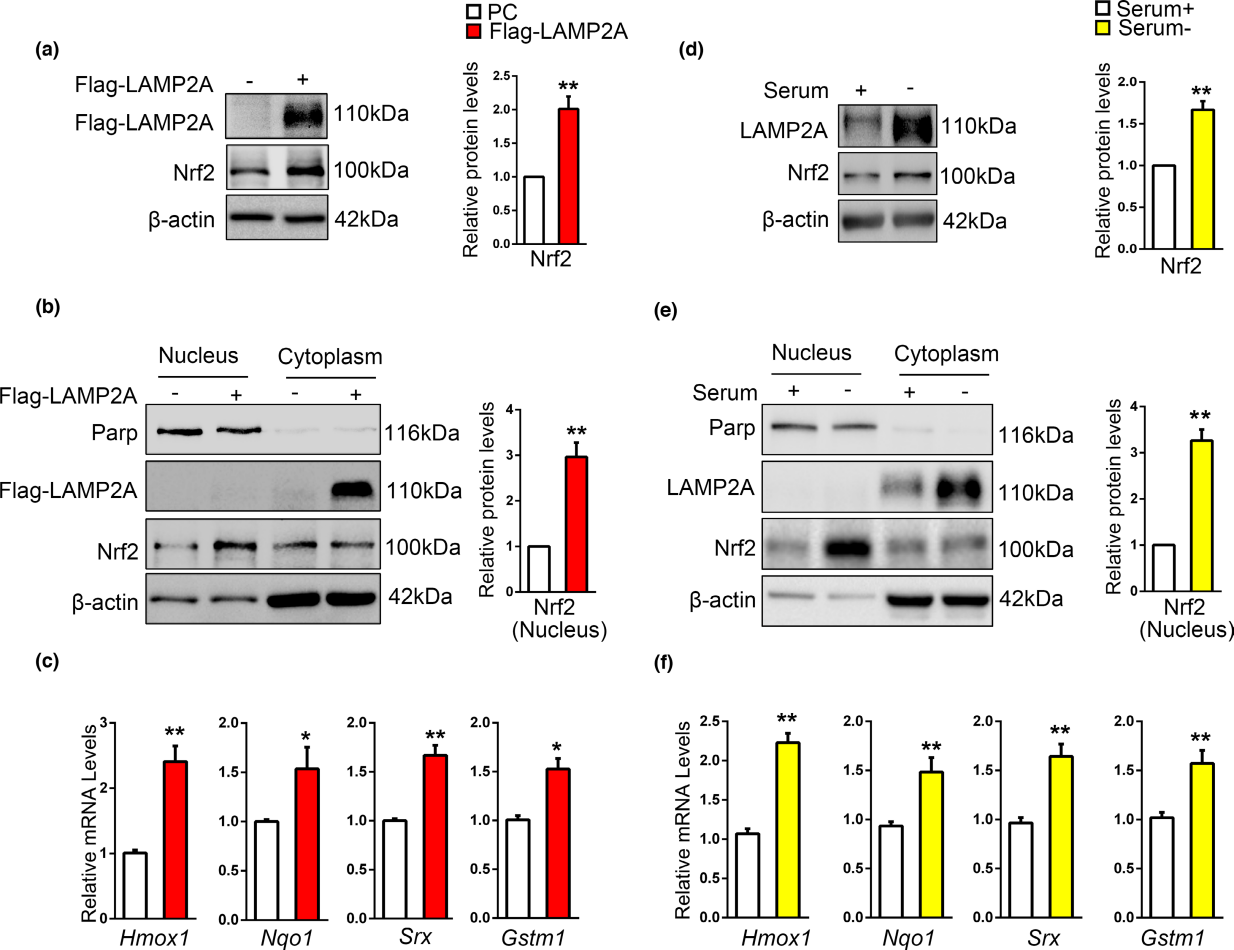
CMA increases the level of Nrf2 protein and its transcriptional activity. (a). The effect of LAMP2A on endogenous Nrf2 protein level. SN4741 cells were transfected with Flag‐LAMP2A plasmids for 48 h. The cell lysates were analyzed by immunoblot with the indicated antibodies. Right panel shows the relative Nrf2 level normalized to β‐actin (Student's *t* test, mean ± SEM, *n* = 3 independent experiments, **p < 0.01). (b). The effect of LAMP2A on the level of Nrf2 in the nucleus. SN4741 cells were treated as in (a). The nuclear or cytoplasmic fractions were prepared for analysis. Right panel shows the relative Nrf2 level normalized to β‐actin (Student's *t* test, mean ± SEM, *n* = 3 independent experiments, **p < 0.01). (c). The effect of LAMP2A on the expression of Nrf2 target genes. SN4741 cells were transfected with Flag‐LAMP2A for 48 h. mRNA was isolated for qPCR. The mRNA level was normalized to that of *β*‐*actin* (Student's *t* test, mean ± SEM, *n* = 6 independent experiments, *p < 0.05, **p < 0.01). (d). The effect of serum deprivation on endogenous Nrf2 protein level. SN4741 cells were maintained in serum‐free media for 48 h. The cell lysates were analyzed by immunoblot with the indicated antibodies. Right panel shows the relative Nrf2 level normalized to β‐actin (Student's *t* test, mean ± SEM, *n* = 3 independent experiments, **p < 0.01). (e). The effect of serum deprivation on the level of Nrf2 in the nucleus. SN4741 cells were treated as in (d). The nuclear or cytoplasmic fractions were prepared for analysis. Right panel shows the relative Nrf2 level normalized to β‐actin (Student's *t* test, mean ± SEM, *n* = 3 independent experiments, **p < 0.01). (f). The effect of serum deprivation on the expression of Nrf2 target genes. SN4741 cells were treated as in (d), mRNA was isolated for qPCR. The mRNA level was normalized to that of *β*‐*actin* (Student's *t* test, mean ± SEM, *n* = 6 independent experiments, *p < 0.05)

SN4741 cells, a mouse midbrain DA progenitor cell line, were used to investigate the relationship between Nrf2 and CMA. As the activity of CMA correlates closely with the level of LAMP2A (Cuervo & Dice, 1996), cells were transiently transfected with Flag‐LAMP2A plasmid for 48 h to activate CMA. Immunoblot and qPCR were employed to detect the levels of Nrf2 protein and mRNA. Results revealed that LAMP2A overexpression upregulated the level of Nrf2 protein but not its mRNA (Figure 1a and Figure S1a). These data suggest that LAMP2A induces Nrf2 accumulation likely via posttranscriptional mechanism. Part of the process of Nrf2 activation involves its translocation into the nucleus. We prepared nuclear and cytoplasmic fractions after LAMP2A overexpression and showed that LAMP2A caused a significant increase of Nrf2 in the nucleus (Figure 1b). To confirm whether Nrf2 was transcriptionally active, we measured mRNA levels of representative Nrf2 target genes involved in antioxidative response including *Hmox1*, *Nqo1*, *Srx*, and *Gstm1* by qPCR. LAMP2A overexpression significantly induced their expression (Figure 1c). Long‐term serum deprivation is another method for activating CMA. SN4741 cells were treated with serum deprivation for 48 h, and immunoblot results showed that LAMP2A is upregulated significantly (Figure 1d), indicating CMA was activated. And the results about Nrf2 were similar to that of LAMP2A overexpression (Figure 1d–f and Figure S1b). We next investigated the effect of inhibiting CMA by transfection of siRNA targeting LAMP2A on Nrf2. However, our analysis showed that under basal condition, LAMP2A knockdown did not alter the level of total or nuclear Nrf2 protein or its targeted mRNA levels (Figure S1c–e). Taken together, these data suggest that activating CMA could increase Nrf2 protein level and transcriptional activity under basal condition.

### 2.2 CMA stabilizes Nrf2 through decreasing the Nrf2 ubiquitination level

**FIGURE 2:**
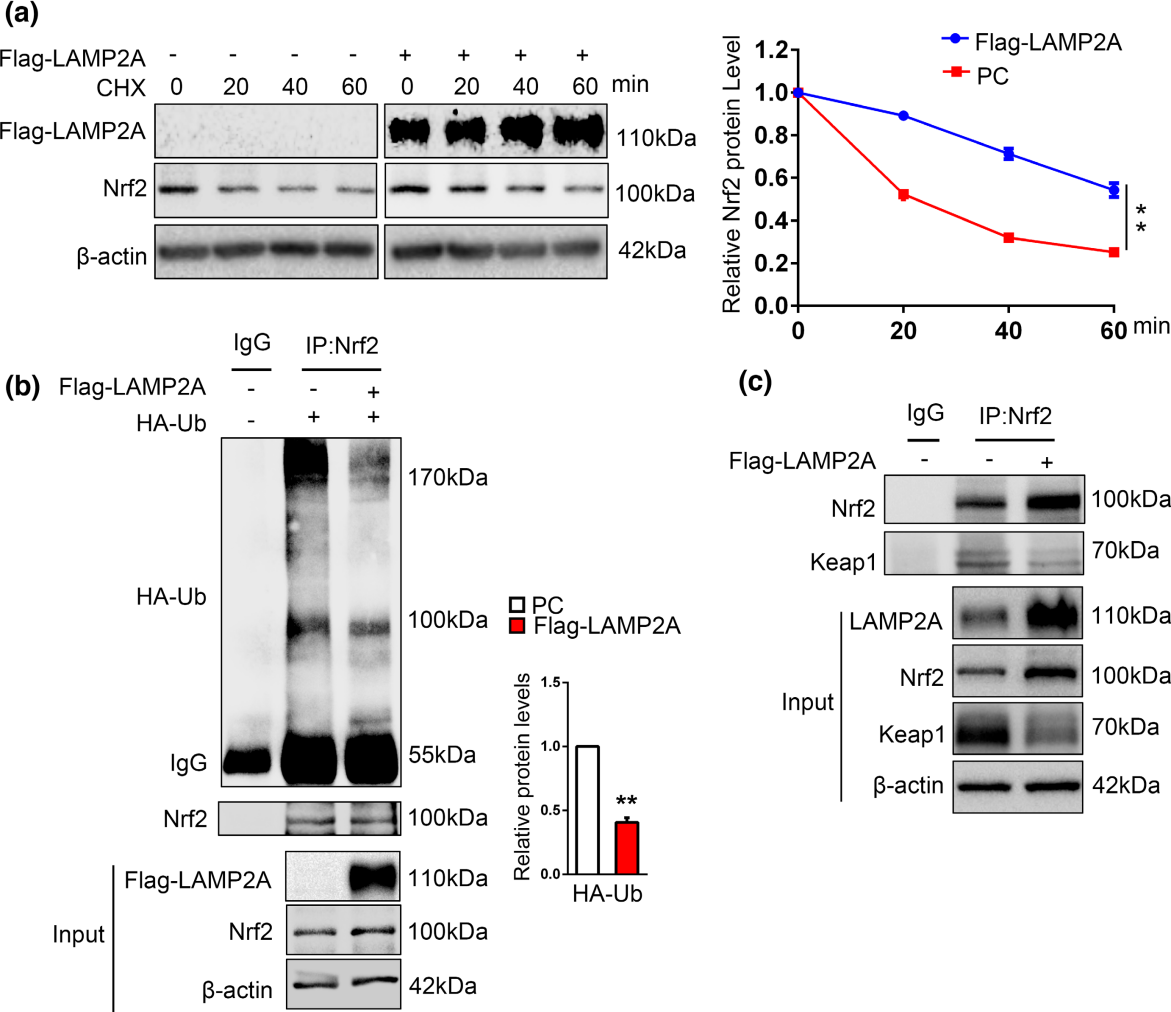
CMA stabilizes Nrf2 through decreasing Nrf2 ubiquitination. (a). Change of Nrf2 half‐life by LAMP2A. SN4741 cells were transfected with the Flag‐LAMP2A plasmid for 48 h and treated with 100 µg/ml CHX for the indicated times. Cells were harvested for immunoblotting with indicated antibodies. Right panel shows the quantification of the Nrf2 protein level normalized to β‐actin. (Two‐way ANOVA followed by Sidak's multiple comparisons test, mean ± SEM, *n* = 3 independent experiments, **p < 0.01). (b). The effect of LAMP2A on Nrf2 ubiquitination. SN4741 cells were transfected with the Flag‐LAMP2A and HA‐ubiquitin (ub) plasmids for 48 h and treated with 10 μM MG132 for another 5 h. The ubiquitination of Nrf2 was determined by immunoprecipitation of Nrf2 and subsequent immunoblot with anti‐HA antibody. Right panel shows the quantification of the HA‐ub protein level normalized to IgG. The input represents 10% of total cell lysates (Student's *t* test, mean ± SEM, *n* = 3 independent experiments, **p < 0.01). (c). The effect of LAMP2A on the interaction between Nrf2 and Keap1. SN4741 cells were transfected with the Flag‐LAMP2A plasmids for 48 h. Lysates were immunoprecipitated with IgG or anti‐Nrf2 antibodies and subsequently immunoblotted with anti‐Keap1 antibody. The input represents 10% of total cell lysates

Our above findings suggest that LAMP2A might elevate Nrf2 protein by regulating its stability. To test this, we treated SN4741 cells with protein synthesis inhibitor cycloheximide (CHX). The Nrf2 protein level decreased gradually in control cells after CHX treatment in a time‐dependent manner, but overexpression of LAMP2A led to a slower degradation of Nrf2 (Figure 2a), suggesting that CMA may stabilize Nrf2. Under basal condition, Nrf2 is known to be continuously ubiquitinated and degraded by proteasome. The stability of Nrf2 negatively correlates with its ubiquitination level (Villeneuve et al., 2010). To determine whether LAMP2A upregulates Nrf2 by modulating its ubiquitination, we measured the level of Nrf2 ubiquitination by overexpressing HA‐ubiquitin in SN4741 cells, immunoprecipitated endogenous Nrf2, and blotted for HA. This analysis showed that Nrf2 was highly ubiquitinated under basal condition and overexpressing LAMP2A greatly reduced the level of ubiquitination (Figure 2b). We also performed similar experiments detecting the effect of Nrf2 when LAMP2A was knocked down. The results showed that LAMP2A knockdown had no significant effect on Nrf2’s stabilization and ubiquitination level (Figure S2a,b). Keap1 protein directly interacts with Nrf2 and regulates its ubiquitination (Kobayashi et al., 2004). Therefore, we analyzed the interaction between Keap1 and Nrf2 under LAMP2A overexpression condition. Our data showed that despite higher level of total Nrf2 being precipitated by anti‐Nrf2 antibody, the amount of Keap1 co‐precipitated with Nrf2 was much less when LAMP2A was overexpressed. Interestingly, the input showed that total Keap1 protein level was reduced following LAMP2A overexpression, correlating with increased Nrf2 (Figure 2c). Therefore, LAMP2A appears to stabilize Nrf2 by reducing the level of Keap1 and thus Nrf2 ubiquitination.

### 2.3 Keap1 is a CMA substrate

**FIGURE 3:**
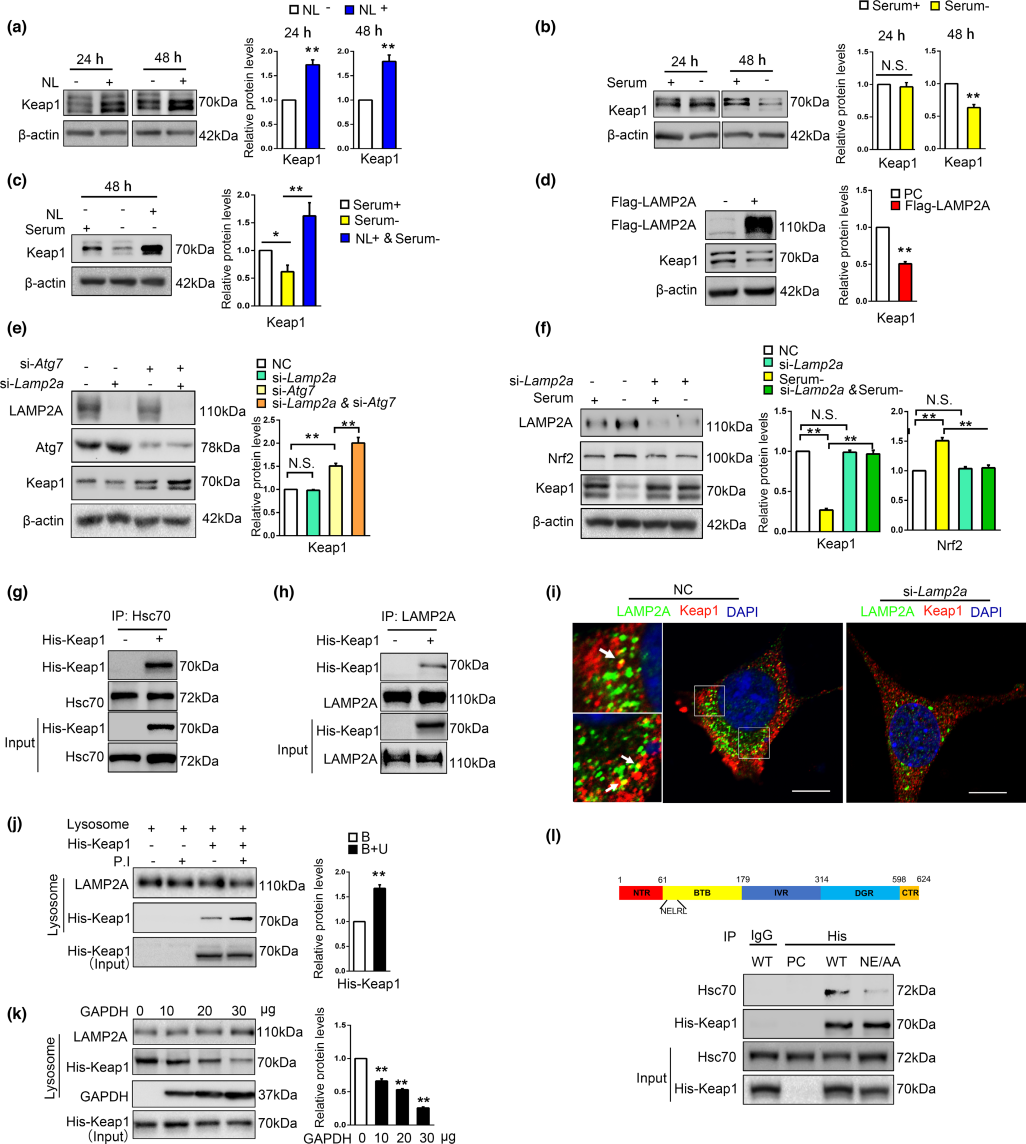
Keap1 is a CMA substrate. (a). The effect of NL on Keap1 protein. SN4741 cells were treated with NL (20 mM NH_4_Cl and 100 mM leupeptin) for indicated time. Cell lysates were analyzed by immunoblot with indicated antibodies. Right panel shows the relative Keap1 level normalized to β‐actin (Student's *t* test, mean ± SEM, *n* = 3 independent experiments, **p < 0.01). (b). The effect of serum deprivation on Keap1 protein level. SN4741 cells were maintained in serum‐free media for 24 and 48 h. The cell lysates were analyzed by immunoblot with the indicated antibodies. Right panel shows the relative Keap1 level normalized to β‐actin (Student's *t* test, mean ± SEM, *n* = 3 independent experiments, **p < 0.01). (c). Change of Keap1 protein level after prolonged serum deprivation and lysosome inhibition. SN4741 cells were treated with serum deprivation and concurrent treatment of NL for 24 and 48 h. Cell lysates were analyzed by immunoblot with indicated antibodies. Right panel shows the relative Keap1 level normalized to β‐actin (Student's *t* test, mean ± SEM, *n* = 3 independent experiments, *p < 0.05, **p < 0.01). (d). The effect of LAMP2A on endogenous Keap1 protein. SN4741 cells were transfected with Flag‐LAMP2A plasmid for 48 h. Cell lysates were analyzed by immunoblot with indicated antibodies. Right panel shows the relative Keap1 level normalized to β‐actin (Student's *t* test, mean ± SEM, *n* = 3 independent experiments, **p < 0.01). (e). The effect of LAMP2A and Atg7 knockdown on the protein level of Keap1 under basal condition. SN4741 cells were transfected with control, si‐*Lamp2a*, or si‐*Atg7* for 48 h. Lysates were analyzed by immunoblot with indicated antibodies. Right panel shows the relative Keap1 level normalized to β‐actin (Student's *t* test, mean ± SEM, *n* = 3 independent experiments, **p < 0.01). (f). The effect of LAMP2A knockdown on serum deprivation‐induced change of Keap1. SN4741 cell were transfected with si‐*Lamp2a* and treated with serum deprivation for 48 h. The cell lysates were analyzed by immunoblot with indicated antibodies. Right panel shows the relative Keap1 and Nrf2 level normalized to β‐actin (Student's *t* test, mean ± SEM, *n* = 3 independent experiments, **p < 0.01). (g). The interaction between exogenous His‐Keap1 and Hsc70. SN4741 cells were transfected with control or His‐Keap1. Co‐IP assays were carried out with an anti‐Hsc70 antibody for IP and an anti‐His antibody for IB. The input represents 10% of total cell lysates. (h). The interaction between exogenous His‐Keap1 and LAMP2A. SN4741 cells were transfected with control or His‐Keap1. Co‐IP assays were carried out with an anti‐LAMP2A antibody for IP and an anti‐His antibody for IB. The input represents 10% of total cell lysates. (i). Analysis of the interaction between Keap1 and LAMP2A by immunofluorescence assay. Keap1 and LAMP2A were stained as red (Alexa Fluor 594) and green (Alexa Fluor 488), respectively. Representative images are shown and the areas of interest are enlarged. The colocalization of Keap1 and LAMP2A is indicated by a yellow color (arrow). Cells treated with si‐*Lamp2a* were used as a negative control (right graph). Scale bar: 10 μm. (j). In vitro binding and uptake assay of Keap1 by lysosomes. The purified lysosomes with or without a cocktail of P.I (P.I, protease inhibitors) were incubated with purified GST‐Hsc70 and the lysates of SN4741 cells overexpressing His‐Keap1 for 30 min at 37℃. After washing, the presence of His‐Keap1 was determined by immunoblotting. The presence of P.I should block lysosomal degradation, and thus, such conditions should measure both Keap1 binding to and taken up by lysosomes, whereas in the absence of P.I, this assay only measures Keap1 binding to lysosomes. The input represents 10% of total cell lysates. Right panel shows the relative Keap1 level normalized to LAMP2A (B: binding; U: uptake) (Student's *t* test, mean ± SEM, *n* = 3 independent experiments, **p < 0.01). (k). The effect of a known CMA substrate GAPDH on the association of Keap1 with lysosomes. The experiment was carried out as in (j) with or without increasing concentrations of GAPDH for 30 min at 37℃. Right panel shows the relative Keap1 level normalized to LAMP2A (Student's *t* test, mean ± SEM, *n* = 3 independent experiments, **p < 0.01). (l). Identification of KFERQ motif required for Keap1 and Hsc70 interaction. Top graph: domain structure of Keap1 protein. Bottom: Lysates of HEK293T cells transfected with wild‐type (WT) and mutated (NE/AA) His‐Keap1 were immunoprecipitated with an anti‐His antibody and immunoblotted with indicated antibodies

Our above results indicated that Keap1 may be a potential substrate of CMA. Previous study reported that Keap1 is targeted to lysosome for degradation, independent of proteasome. To prove it, MG132, the proteasome inhibitor, and a combination of NH_4_Cl and leupeptin (NL), the lysosomal hydrolase inhibitor were used to treat SN4741 cells. Immunoblot results revealed that MG132 caused significant accumulation of ubiquitinated proteins, but had little effect on the levels of Keap1 (Figure S3a). And NL caused a significant accumulation of Keap1 (Figure 3a). Then, to determine whether Keap1 is a CMA substrate, we subjected SN4741 cells to prolonged serum deprivation, which has been shown to activate CMA effectively (Gao et al., 2014). Keap1 level was reduced following 48‐h but not 24‐h serum deprivation (Figure 3b), and NL could reverse the reduction of Keap1 (Figure 3c). Consistent with above findings, LAMP2A overexpression for 48 h significantly decreased the Keap1 level under basal condition (Figure 3d). However, LAMP2A knockdown had no obvious influence on Keap1 level (Figure S3b). It has been reported that Keap1 could be degraded through macroautophagy and macroautophagy is compensatorily activated when CMA is blocked under basal condition (Massey et al., 2005; Taguchi et al., 2012), so it is possible that increased Keap1 may be timely removed by activated macroautophagy in LAMP2A‐knockdown cells. Atg7 is the key factor for macroautophagy process. We knocked down LAMP2A with or without concurrent Atg7 knockdown and tested the protein level of Keap1. The results showed that in contrast to cells with normal Atg7 expression, interfering with LAMP2A expression elicited a more fold of increase of Keap1 level in Atg7 knocked‐down cells (Figure 3e). In line with this, after 48‐h serum deprivation, when macroautophagy activity has been shown to greatly decreased and CMA becomes activated, knockdown of LAMP2A significantly reversed the reduction of Keap1 as well as the upregulation of Nrf2 (Figure 3f; Fuertes et al., 2003). Thus, apart from macroautophagy, CMA is another important pathway regulating Keap1 turnover.

CMA regulator Hsc70 interacts with substrate proteins containing KFERQ‐like motifs (Dice, 1990). We overexpressed Keap1 and incubated the cell lysates with purified GST‐Hsc70 in a GST pull‐down assay. The analysis showed that Keap1 interacted with GST‐Hsc70 (Figure S3c). Co‐immunoprecipitation data also revealed that Keap1 associated with endogenous Hsc70 (Figure 3g and Figure S3d). CMA substrates are delivered by Hsc70 to lysosome membrane and interact with LAMP2A to be internalized into lysosomal lumen. The co‐immunoprecipitation and immunofluorescence co‐labeling assays confirmed the interaction between Keap1 and LAMP2A (Figure 3h and Figure S3e,i). We then conducted lysosomal binding and uptake assay, a gold standard for confirming a protein as CMA substrate (Kaushik & Cuervo, 2012). SN4741 cell lysates with His‐Keap1 overexpressed were incubated with lysosomes purified from rat liver with or without protease inhibitors (P.I). The results showed that Keap1 could bind with and be taken up into lysosomes (Figure 3j). Besides, co‐incubation of Keap1 lysates with the increased amount of purified GAPDH, a known CMA substrate protein, dose‐dependently reduced the amounts of Keap1 associated with and taken up by the lysosomes (Figure 3k). We searched the KFERQ‐like motif in Keap1 using the “KFERQ finder” (Kirchner et al., 2019), which identified NELRL^68−72^ sequence as a candidate KFERQ‐like motif in Keap1. Besides, the adjacent LRLSQ^70−74^ also was identified as a phosphorylation‐generated motif (S to D/E). We then mutated N^68^ and E^69^ to alanine (NE/AA) and showed by immunoprecipitation analysis that Keap1 NE/AA mutant had significantly lower affinity with Hsc70 compared with wild‐type (WT) Keap1 (Figure 3l). However, SQ/AA mutation showed no obvious disruption on the interaction between Keap1 and Hsc70 (Figure S3h). These data experimentally support NELRL^68−72^ as a KFERQ‐like motif. Together, our findings establish Keap1 as a CMA substrate.

### 2.4 CMA degrades Keap1 in response to 6‐OHDA in SN4741 cells

**FIGURE 4:**
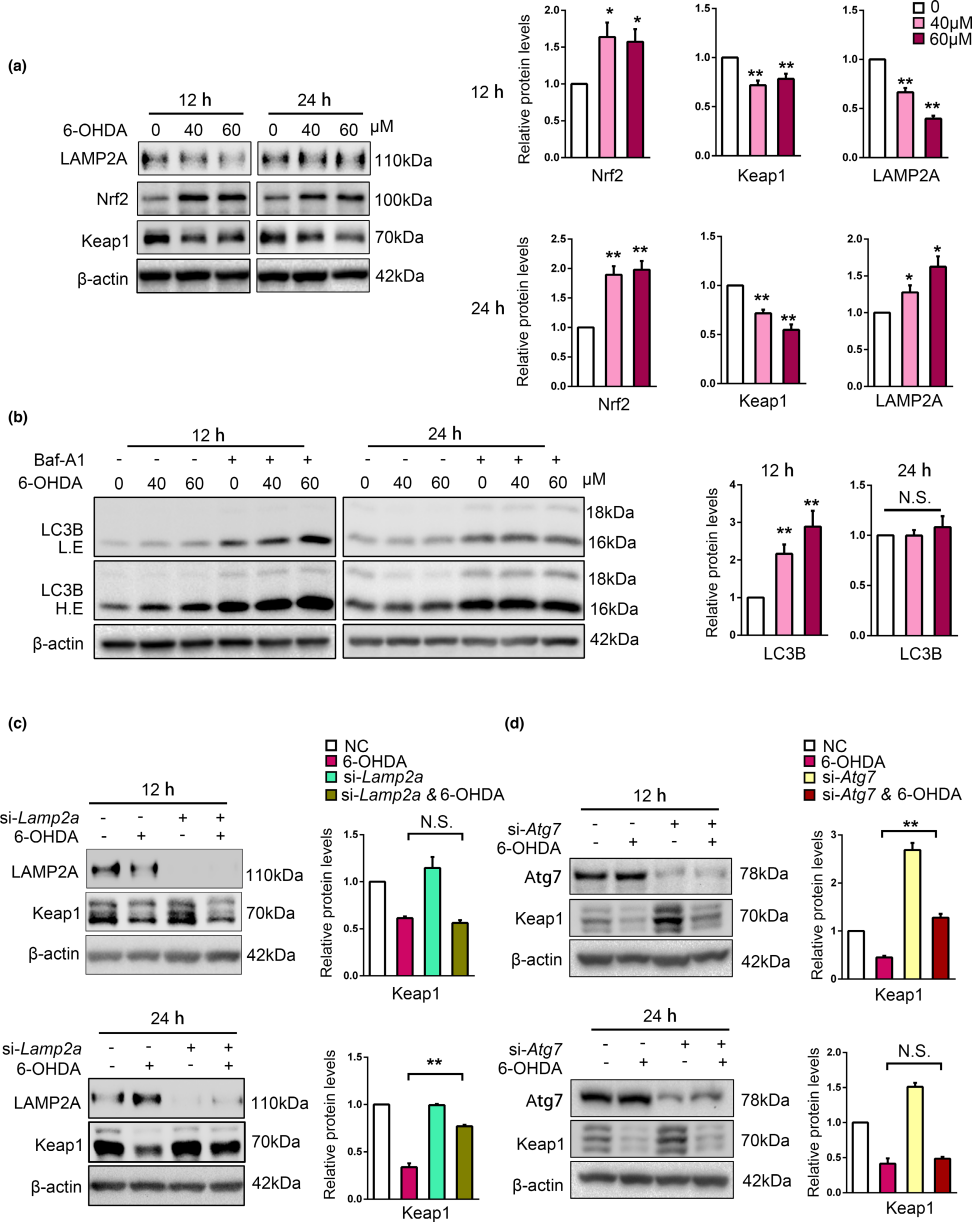
6‐OHDA induces CMA‐dependent degradation of Keap1 in SN4741 cells. (a). The effects of 6‐OHDA treatment on Nrf2, Keap1, and LAMP2A. SN4741 cells were treated with 40 or 60 μM 6‐OHDA for 12 or 24 h. The cell lysates were analyzed by immunoblot with indicated antibodies. Right panel shows the relative protein level normalized to β‐actin (Student's *t* test, mean ± SEM, *n* = 3 independent experiments, **p < 0.01). (b). The effect of 6‐OHDA on LC3B. SN4741 cells were exposed to 40 or 60 μM 6‐OHDA with or without 100 nM Baf‐A1 for 12 or 24 h. The cell lysates were analyzed by immunoblot with indicated antibodies. Right panel shows the relative LC3B level normalized to β‐actin (Student's *t* test, mean ± SEM, *n* = 3 independent experiments, **p < 0.01, N.S., no significance). (c). The effect of LAMP2A knockdown on Keap1 level following 6‐OHDA treatment. SN4741 cells were transfected with control or si‐*Lamp2a* and then treated with 60 μM 6‐OHDA for 12 and 24 h. The cell lysates were analyzed by immunoblot with indicated antibodies. Right panel shows the relative Keap1 level normalized to β‐actin (Student's *t* test, mean ± SEM, *n* = 3 independent experiments, **p < 0.01). (d). The effect of Atg7 knockdown on Keap1 level following 6‐OHDA treatment. SN4741 cells were transfected with control or si‐*Lamp2a* and then treated with 60 μM 6‐OHDA for 12 and 24 h. The cell lysates were analyzed by immunoblot with indicated antibodies. Right panel shows the relative Keap1 level normalized to β‐actin (Student's *t* test, mean ± SEM, *n* = 3 independent experiments, **p < 0.01)

Keap1‐mediated regulation of Nrf2 is the major mechanism by which Nrf2 responds to oxidative stress or electrophilic stress conditions (Kensler et al., 2007; Yamamoto et al., 2018). To investigate the role of CMA‐Keap1‐Nrf2 axis in response to oxidative stress, we treated SN4741 cells with different doses of 6‐OHDA, a commonly used neurotoxin to induce oxidative damage specifically in DA neurons (David Blum et al., 2001), and determined the level of Nrf2. We found that Nrf2 level was increased after 12‐ and 24‐h treatment of 40 and 60 μM 6‐OHDA, accompanied with reduced Keap1 protein level (Figure 4a). LAMP2A level was reduced after 12 h 6‐OHDA treatment but significantly increased at 24 h (Figure 4a). Besides, we used another oxidative stress inducer, H_2_O_2_ to treat SN4741 cell for 12 and 24 h and repeated related experiments (Figure S4). The results showed that Nrf2 and LAMP2A level increased upon H_2_O_2_ treatment, similar to the observation under 6‐OHDA treatment. By contrast, assessment of LC3BII level after bafilomycin A1 treatment indicated that 6‐OHDA increased macroautophagy flux at 12 h and this induction was largely lost by 24 h (Nie et al., 2016; Figure 4b). To detect which type of autophagy is responsible for the degradation of Keap1 under long‐term oxidative stress, we transfected SN4741 cells with si‐*Lamp2a* or si‐*Atg7* to inhibited CMA or macroautophagy, respectively, and then treated cells with 6‐OHDA for either 12 or 24 h. Consistent with the time‐dependent dynamic change of activity of these two manners of autophagy, we found that LAMP2A knockdown effectively attenuated the reduction of Keap1 in 24‐h 6‐OHDA treatment, but not 12 h (Figure 4c). Besides, Atg7 knockdown could increase Keap1 level in basal condition, and partly reversed the reduction of Keap1 in 12 h, but could not significantly block the degradation of Keap1 in 24‐h 6‐OHDA treatment (Figure 4d). These data suggest that 6‐OHDA‐induced oxidative stress regulates macroautophagy and CMA with differential kinetics, and Keap1 is primarily degraded by CMA in long‐term oxidative stress condition induced by 6‐OHDA, which is consistent with CMA as the main mechanism regulating Keap1‐Nrf2 pathway during prolonged stress.

### 2.5 Interfering CMA disturbs Nrf2 antioxidative response

**FIGURE 5:**
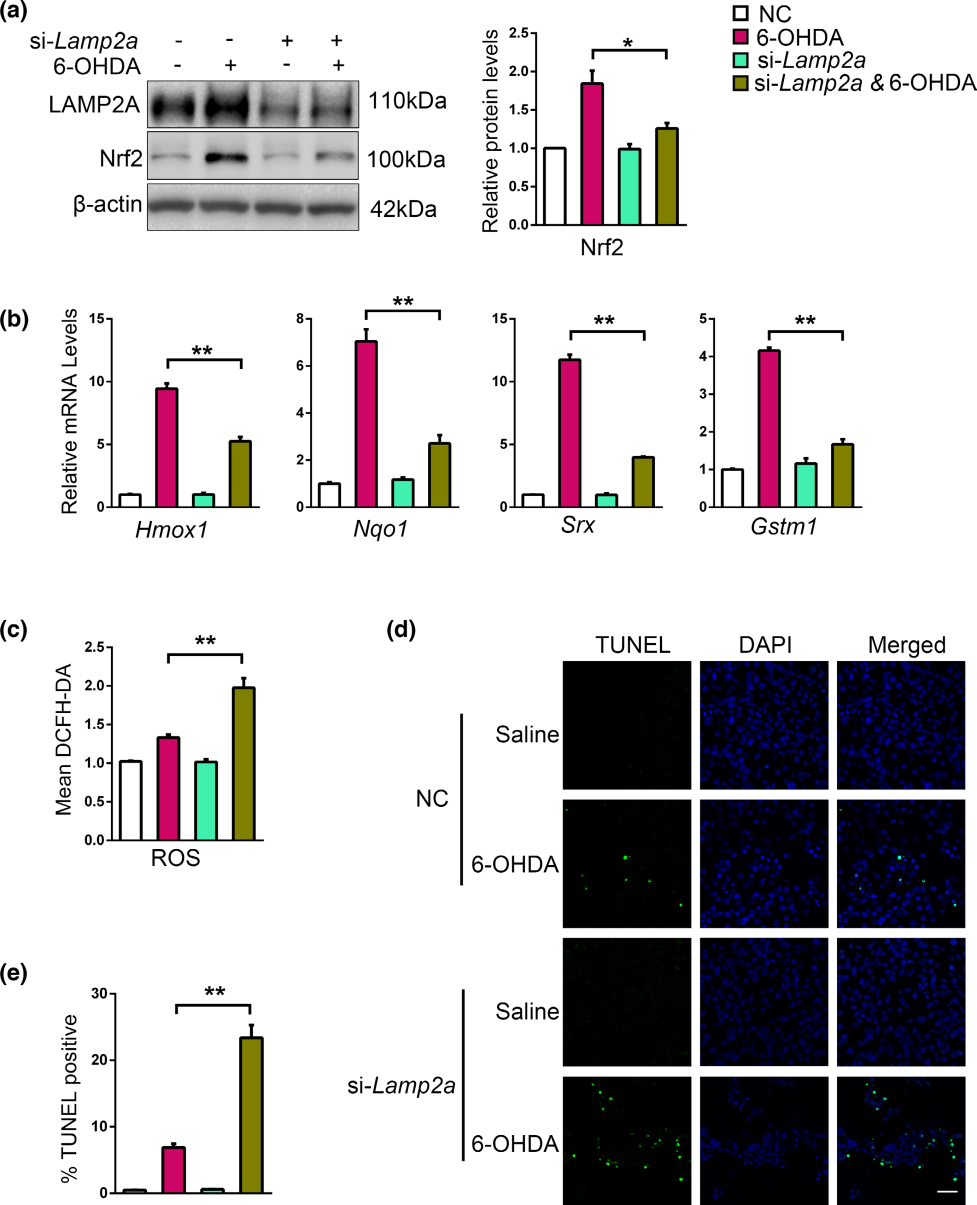
Interfering with CMA disturbs Nrf2 antioxidative response. (a). The effect of LAMP2A knockdown on 6‐OHDA‐induced change of Nrf2. SN4741 cells were transfected with control or si‐LAMP2A and treated with 60 μM 6‐OHDA for 24 h. The cell lysates were analyzed by immunoblot with indicated antibodies. Right panel shows the relative Nrf2 level normalized to β‐actin (Student's *t* test, mean ± SEM, *n* = 3 independent experiments, *p < 0.05). (b). The effect of LAMP2A knockdown on 6‐OHDA‐induced change of Nrf2 target gene expression. mRNA in control and LAMP2A knockdown SN4741 cells with 6‐OHDA treatment. SN4741 cells were treated as described in (a) and mRNAs of Nrf2 target genes were measured by qPCR. The indicated mRNA level was normalized to β‐actin (Student's *t* test, mean ± SEM, *n* = 6 independent experiments, **p < 0.01). (c). The effect of LAMP2A knockdown on ROS following 6‐OHDA treatment. SN4741 cells were treated as described in a, incubated with H2DCF‐DA for 30 min, washed 2 times with prewarmed PBS, and collected for counting. The same number of cells was used for analysis with microplate reader (Student's *t* test, mean ± SEM, *n* = 3 independent experiments, *p < 0.05). (d). The effect of LAMP2A knockdown on 6‐OHDA‐induced cell death. SN4741 cells were treated as described in a. TUNEL assay was performed to determine the cell viability (bar = 50 μm). The statistical analysis is shown in (e) (Student's *t* test, mean ± SEM, *n* = 3 independent experiments, *p < 0.05)

As 6‐OHDA‐induced Keap1 degradation requires CMA, we next tested if CMA is required for Nrf2‐dependent antioxidative response. Immunoblot results showed that knockdown of LAMP2A in SN4741 cells significantly attenuated 6‐OHDA‐induced increase of Nrf2 protein (Figure 5a). 6‐OHDA treatment stimulated the expression of several Nrf2‐dependent antioxidative genes including *Hmox1*, *Nqo1*, *Srx*, and *Gstm1*. Their expression was significantly reduced when LAMP2A was knocked down (Figure 5b). Nrf2‐mediated response clears ROS and protects cells from ROS‐induced death. LAMP2A knockdown abrogated this ability and led to a significant accumulation of ROS and increase in cell death (Figure 5c–e). These data support that CMA is necessary for the efficient antioxidative response of Nrf2.

### 2.6 The positive feedback loop between Nrf2 and CMA

**FIGURE 6:**
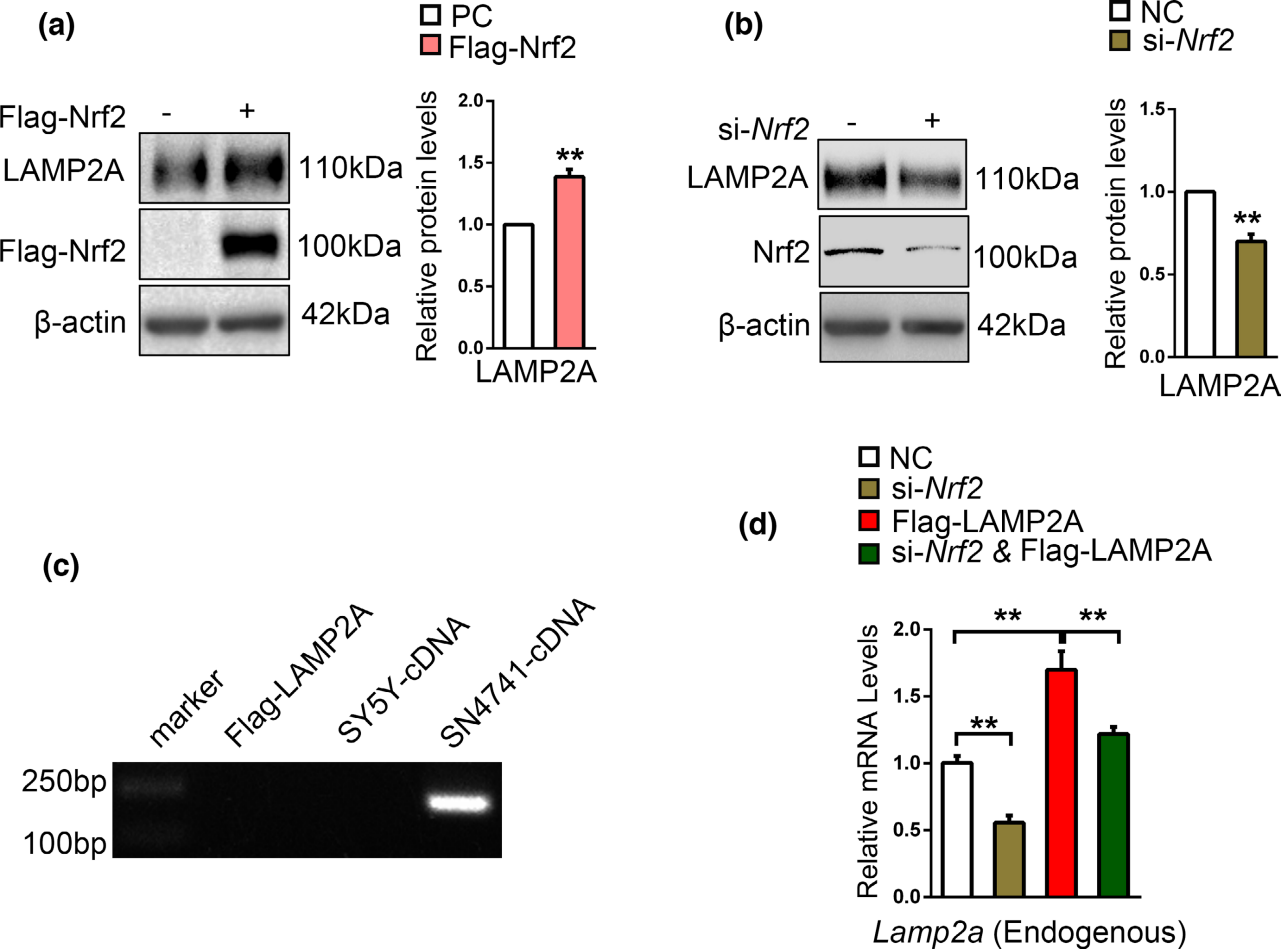
Nrf2 increases the level of LAMP2A and a CMA‐Nrf2‐positive feedback model. (a). The effect of Nrf2 on the level of endogenous LAMP2A protein. SN4741 cells were transfected with Flag‐Nrf2 plasmid for 48 h. The cell lysates were analyzed by Western blot with indicated antibodies. Right panel shows the relative Nrf2 level normalized to β‐actin (Student's *t* test, mean ± SEM, *n* = 3 independent experiments, **p < 0.01). (b). The effect of Nrf2 Knockdown on the level of endogenous LAMP2A protein. SN4741 cells were transfected with si‐Nrf2 for 48 h. The cell lysates were analyzed by Western blot with indicated antibodies. Right panel shows the relative Nrf2 level normalized to β‐actin (Student's *t* test, mean ± SEM, *n* = 3 independent experiments, **p < 0.01). (c). Verification of the primers specific for mouse *Lamp2a* mRNA. Flag‐LAMP2A plasmids, cDNA from SH‐SY5Y cells and SN4741 cells were amplificated by PCR and DNA electrophoresis was carried out to determine primer specificity for mouse *Lamp2a* mRNA. (d). The effect of Nrf2 knockdown on the level of endogenous *Lamp2a* mRNA following overexpression of human Flag‐LAMP2A. SN4741 cells were transfected with as indicated for 48 h. The level of mouse *Lamp2a* mRNA was determined by qPCR. The endogenous *Lamp2a* mRNA level was normalized to that of *β*‐*actin*. (Student's *t* test, mean ± SEM, *n* = 6 independent experiments, **p < 0.01)

Above results indicate that CMA activates Nrf2 through degrading Keap1. Nrf2 has been reported to transcriptionally controls LAMP2A expression and consequently actives CMA (Pajares et al., 2018). To validate it in our model system, we transfected SN4741 cells with Flag‐Nrf2 plasmids or siRNA specific for Nrf2, respectively, and blotted LAMP2A. This analysis showed that LAMP2A protein level correlated positively with the level of Nrf2 (Figure 6a,b). Since LAMP2A and Nrf2 may regulate each other, we tested if exogenous LAMP2A could increase endogenous LAMP2A through Nrf2. Our Flag‐LAMP2A plasmid was cloned from human LAMP2A cDNA, and SN4741 is a progenitor cell line derived from mouse embryonic midbrain. Even though human LAMP2A is functionally interchangeable with the mouse protein (Cuervo & Dice, 2000), their gene sequences have species differences. We designed a pair of special qPCR primers specific for mouse LAMP2A and DNA electrophoresis result verified the primer's specificity to SN4741 cells, not Flag‐LAMP2A plasmid or SH‐SY5Y cells (a human neuroblastoma cell line; Figure 6c). We overexpressed human LAMP2A, knocked down Nrf2 in SN4741 cells, and measured mouse LAMP2A mRNA by qPCR. Our results reveled that exogenous LAMP2A substantially increased the level of endogenous LAMP2A mRNA and the effect was blocked in Nrf2 knocked‐down cells (Figure 6d). *Lamp2a*, *Lamp2b*, and *Lamp2c* isoforms originate from alternative splicing of the *Lamp2* gene, which is cell‐specific (Eskelinen et al., 2005). And study supports that LAMP2A is the only LAMP2 variant required for CMA (Massey et al., 2006). The expression of *Lamp2b* and *Lamp2c* was not significantly changed in SN4741 cells, compared with *Lamp2a* under same treatment (Figure S5a). The reason might be that these two isoforms are regulation‐ and cell‐specific expression. To exclude whether LAMP2A upregulation is a result of overall expansion of lysosomes, we detected the expression of *Lamp1* and *Ctsb* via qPCR, which are the transcriptional target of TFEB and reflect the overall lysosome content. The results showed no obvious change of the mRNA level of *Lamp1* and *Ctsb* (Figure S5b). Taken together, our findings suggest a positive feedback regulation network between CMA and Nrf2.

## 3 DISCUSSION

CMA is involved in multiple functions important for neuronal homeostasis including protecting mitochondrial function (Wang et al., 2016) and inhibiting excessive endoplasmic reticulum stress (Li et al., 2017). The primary mechanism known for CMA to alleviate oxidative stress is to remove oxidatively damaged substrates individually and thereby maintain protein quality control (Anguiano et al., 2013; Issa et al., 2018). In this study, we provide evidence to show that CMA actively participates in the master regulatory program that controls the cellular antioxidative response at a much larger scale. CMA, in response to oxidative signal, directly degrades Keap1 to stabilize Nrf2 and facilitate its nuclear translocation, which is essential for the transcriptional activation of multiple antioxidant genes. Since Nrf2 also stimulates the expression of key CMA regulator LAMP2A, CMA‐Nrf2 forms a positive feedback loop to augment their antioxidative stress responses.

Mechanistically, we have demonstrated that Keap1, the critical regulatory factor of Nrf2, is directly targeted by CMA. Under basal condition, augmentation of CMA by LAMP2A overexpression was sufficient to degrade Keap1 and activate Nrf2. By contrast, inhibition of CMA by LAMP2A knockdown did not alter overtly the levels of Keap1 and Nrf2 in the absence of stress signal. One interpretation of such findings is that CMA does not play a major role in regulating Keap1 level under basal condition. CMA actively engages Keap1 upon signaling, and this is particularly important for cells to mount an appropriate level of Nrf2 response adequate for the nature and stress of the signals.

CMA, macroautophagy, and ubiquitin proteasome system (UPS) processes are often intertwined in a complex cellular response and share some of their regulatory substrates (Kiffin et al., 2004; Mak et al., 2010; Massey et al., 2005). For instance, alpha‐synuclein has been found to be degraded through UPS, macroautophagy, and CMA. Studies have indicated that Keap1 can be degraded through p62‐dependent macroautophagy in hepatocytes and fibroblasts (Bae et al., 2013; Wang, Chen et al., 2019). Therefore, inhibition of CMA can lead to compensatory degradation of Keap1 through macroautophagy under normal conditions. Consistent with published data (Zhang et al., 2005), our result indicated that proteasome does not participate in the degradation of Keap1 under basal condition. Further studies are required to investigate whether proteasome participates in degradation of Keap1 in other conditions or types of cells. Our data indicate that 6‐OHDA activates macroautophagy and CMA sequentially. Interestingly, our data showed that while the elevation of Nrf2 level was robust and coincided with the activation of macroautophagy and CMA, the level of Keap1 remained relatively consistent during macroautophagy activation but decreased dose‐dependently, when CMA was activated. This would be consistent with CMA as the main mechanism regulating Keap1‐Nrf2 pathway during prolonged stress. Whether this differential and sequential activation of macroautophagy and CMA may reflect the distinct roles of these pathways in the typically progressive and prolonged course of neurodegenerative process remains to be clarified.

We used in vitro lysosomal association and uptake assays to establish Keap1 as a CMA substrate. Kirchner et al. (2019) previously developed a free Web‐based resource (KFERQ finder) for direct identification of KFERQ‐like motifs in any protein sequence. Using this useful tool, we identified NELRL^68−72^ as the canonical KFERQ‐like motif of Keap1. Besides, the KFERQ finder also provides several additional noncanonical motifs generated through phosphorylation in Keap1 sequence. It might explain that mutation of NE^68−69^ to AA, although greatly reducing the interaction between Keap1 and Hsc70, did not completely abolish their binding (Figure 3l). These noncanonical motifs’ effects on the interaction between Keap1 and Hsc70, as well as Keap1’s degradation, need further research.

Our study demonstrates that CMA is necessary for Nrf2‐mediated antioxidative response. It is interesting to note that Nrf2 has been reported to transcriptionally regulate LAMP2A and consequently activate CMA (Pajares et al., 2018), and we showed that LAMP2A positively regulates its mRNA via Nrf2 in SN4741 cells. Together, these findings reveal a positive feedback regulation network between CMA and Nrf2. Our model proposes that mild long‐term oxidative stress activates CMA, which stimulates the transcriptional activity of Nrf2. Increased Nrf2, in turn, upregulates *Lamp2a* gene expression, and this further augments CMA activity. This positive feedback loop should allow cells to mount the needed antioxidative response quickly. Interestingly, since macroautophagy can stabilize Nrf2, Nrf2 upregulates the transcription of genes related to macroautophagy such as p62, ULK1, Atg2b, Atg5, and Atg7 (Jain et al., 2010; Pajares et al., 2016). Therefore, there may also be a positive feedback loop between macroautophagy and Nrf2. The presence of these double feedback loops indicates a tight and broad integration of autophagy and Nrf2 and places Nrf2 in a unique and critical regulatory node coordinating lysosomal function and antioxidative response. Changes in autophagy, oxidative stress, and Nrf2 have been noted in aging and neurodegenerative conditions. Loss of such a coordination may contribute to many pathogenic processes.

## 4 EXPERIMENTAL PROCEDURES

### 4.1 Cell culture

SN4741 cells, a mouse embryonic substantia nigra‐derived cell line (Son et al., 1999), were cultured at 33℃ with 5% CO_2_ in DMEM supplemented with 10% fetal bovine serum (FBS), 1% D‐glucose, and 120 mM L‐glutamine. Human embryonic kidney 293T cells (HEK293T) and SH‐SY5Y cells were grown in DMEM supplemented with 10% FBS at 37℃ with 5% CO_2_. A total of 60%–70% confluence was suitable for experiments.

### 4.2 Antibodies and regents

The antibodies used for immunoblot and immunoprecipitation were listed as follows: anti‐LAMP2A (cat # ab125068, Abcam), anti‐LAMP1 (cat # 9091, Cell Signaling Technology), anti‐Hsc70 (cat # ab51052, Abcam), anti‐Nrf2 (cat # 16296‐1‐AP, Proteintech), anti‐Keap1 (cat # 10503‐2‐AP & cat # 60027‐1‐Ig, Proteintech), anti‐β‐actin (cat # AC037, ABclonal), anti‐Ubiquitin (cat # 3933, Cell Signaling Technology), anti‐Flag (cat # 20543‐1‐AP, Proteintech), anti‐HA (cat # 51064‐2‐AP, Proteintech), anti‐His (cat # 66005‐1‐Ig, Proteintech), anti‐GST (cat # AE001, ABclonal), anti‐GAPDH (cat #5174, Cell Signaling Technology), anti‐LC3B (cat # 3868, Cell Signaling Technology), anti‐Atg7 (cat # 8558, Cell Signaling Technology), anti‐VDAC1 (cat # 4661, Cell Signaling Technology), and anti‐Parp (cat # 46D11, Cell Signaling Technology). HRP‐labeled Goat anti‐Mouse IgG (cat # AS003) and Goat anti‐Rabbit IgG (cat # AS014) were purchased from ABclonal. Alexa Fluor 488‐adsorbed goat anti‐rabbit IgG (cat # A11008) and Alexa Fluor 594‐adsorbed goat anti‐mouse IgG (cat # A11032) were purchased from Invitrogen. The chemicals used were listed as follows: NH_4_Cl (Ammonium chloride, cat # A9434, Sigma), Leupeptin (cat # 103476‐89‐7, MP Biomedicals), 6‐OHDA (6‐hydroxydopamine hydrochloride, cat # H4381, Sigma), Baf‐A1 (bafilomycin A1, cat # ab120497, Abcam), MG132 (cat # S2619, Selleck), and CHX (cycloheximide, cat # HY‐12320, MedchemExpress).

### 4.3 Plasmid and small interfering RNA (siRNA) transfection

The Flag‐LAMP2A plasmid was a gift from Dr. Zixu Mao (Department of Pharmacology and Neurology, School of Medicine, Emory University, Atlanta, USA) and the HA‐Ub plasmid was a gift from Dr. Quan Chen (State Key Laboratory of Membrane Biology, Institute of Zoology, Chinese Academy of Sciences, Beijing, China). Flag‐Nrf2 were purchased from GeneChem.

Plasmids expressing His‐Keap1 were generated using PCR and cloned into PCDNA 3.1(‐). Keap1 site‐directed mutagenesis was performed using the QuickMutation™ Site‐Directed Mutagenesis Kit (cat # D0206, Beyotime) according to the manufacturer's instructions. Wild‐type His‐Keap1 was used as a template in the mutagenesis reaction. The mutagenic oligonucleotides designed to produce the desired point mutations were as follows: NE/AA, 5′‐CTGGCTCAGGCGAAGCGCGGCCATGACGCCAAAAGCC‐3′ (forward) and 5′‐GGCTTTTGGCGTCATGGCCGCGCTTCGCCTGAGCCAG‐3′ (reverse); SQ/AA, 5′‐GTCACGTCACAGAGTTGCGCGGCCAGGCGAAGCTCGTTCAT‐3′ (forward) and 5′‐ATGAACGAGCTTCGCCTGGCCGCGCAACTCTGTGACGTGAC‐3′ (reverse).

Small interfering RNA targeting LAMP2A and Nrf2 were purchased from Sangon Biotech, and the sequences were as follows: si‐*Lamp2a*, 5′‐CCAUUGCAGUACCUGACAATT‐3′ (sense) and 5′‐UUGUCAGGUACUGCAAUGGTT (antisense); si‐*Nrf2*, 5′‐CGAGAAGUGUUUGACUUUATT‐3′ (sense), and 5′‐UAAAGUCAAACACUUCUCGTT‐3′ (antisense). Small interfering RNA targeting *Atg7* was purchased from TsingKe Biological Technology, and the sequences were as follows: 5′‐GGAGCAUGCCUAUGAUGAUTT‐3′ (sense) and 5′‐AUCAUCAUAGGCAUGCUCCTT‐3′ (antisense).

Lipofectamine 2000 (cat # 11668019, Invitrogen) was used for transfection experiments following the manufacturer's instructions. And control group cells were transfected with related empty vector plasmids or negative control siRNA.

### 4.4 Western blot

After washed 3 times with ice‐cold PBS, cells were collected by centrifugation and lysed in RIPA buffer (cat # P0013C, Beyotime) containing protease cocktail inhibitor (cat # 539134, Millipore) and Phosphatase inhibitor (cat # 524625, Millipore) for 30 min on the ice. Lysates were sonicated for 5 s 3 times and centrifuged at 13,000 *g* for 10 min. The protein concentration was determined using a BCA protein assay kit (cat # 2322, Thermo). Equal amount of protein from each sample was separated by 8%–12% SDS‐PAGE and transferred to PVDF membranes (cat # 03010040001, Roche). Then, membranes were blocked with 5% fat‐extracted milk at room temperature for 2 h, incubated with primary antibodies overnight at 4°C, washed 3 × 5 min with TBST, and incubated with secondary antibody. For cytoplasm and nuclear separation, the Subcellular and Protein Fractionation Kit (cat # 78840, Thermo) was used for separating the cytoplasm and nuclear protein. The fractionated cellular proteins were used for immunoblotting. Protein bands were visualized using ECL and analyzed by ImageJ.

### 4.5 Co‐immunoprecipitation

Cells were collected and lysed in a lysis buffer (50 mM Tris–HCl, 150 mM NaCl, 1 mM EDTA, 1% Triton X‐100, 2 mM DTT) containing protease cocktail inhibitor (cat # 539134, Millipore) and Phosphatase inhibitor (cat # 524625, Millipore). Cell lysates were incubated with indicated antibody overnight at 4°C, followed by 4‐h A/G‐plus agarose bead (cat # sc‐2003, Santa Cruz Biotechnology) incubation at 4°C. Thereafter, the precipitants were washed 3 times with ice‐cold lysis buffer, and the proteins were eluted from beads by heating in loading buffer. Then, the precipitates were separated by SDS‐PAGE and analyzed by standard Western blot.

### 4.6 GST pull down

BL21 engineered bacteria containing GST or GST‐Hsc70 were cultured in 100 ml LB medium (NaCl 12.5 g/L, typtone 12.5 g/L, and yeast extract 7.5 g/L) at 37℃ in an orbital shaker for 12 h, induced by 1 mM/L IPTG (cat # 420322, Millipore) for another 5 h to induce the expression of GST and GST‐Hsc70 protein, and lysed by PBST (PBS + 1% Triton X). After ultrasonic treatment, the lysates were incubated with glutathione agarose beads for 2 h at 4°C. Then, the beads containing GST or GST‐Hsc70 were collected after washed 3 times with ice‐cold PBST. HEK293T was collected and lysed with RIPA buffer after transfection with vector‐Keap1 plasmid for 48 h. After that, the cell lysates were incubated with beads for another 2 h. The bound proteins were washed and analyzed by standard Western blot.

### 4.7 Lysosome isolation

Lysosome isolation was performed as described previously using lysosome enrichment kit (cat # 89839, Thermo Scientific; Nie et al., 2020). Briefly, male Sprague Dawley rats (200–250 g) were fasted for 24 h and then were sacrificed via anesthetic overdose (CO2). Rats’ livers were harvested and homogenized in the extraction buffer (0.25 M sucrose, 1 mM EDTA, 20 mM HEPES, pH 7.4). The homogenate was centrifuged at 800–1000 *g* for 10 mins and transferred into a clean tube. Then, the collected supernatant was centrifuged at 20,000 *g* for 10 min. Supernatant was removed, and pellet was resuspended with 9% OptiPrep (cat # D1556, Sigma) solution. In a new ultracentrifuge tube, a discontinuous density gradient was prepared by overlapping the OptiPrep gradients in descending concentrations (18%, 16%, 14%, 12%, and 10%). Then, resuspended pellet was carefully layered onto the top of density gradients. The sample was centrifuged at 145,000 *g* for 2 h. The CMA‐activated lysosome (Hsc70 enriched) band is close to the top of the gradient (Figure S3f). Lysosome integrity was assessed by measuring the activity of HEX/β‐hexosaminidase as previously reported (Storrie & Madden, 1990; Figure S3g). Preparations with more than 10% of broken lysosomes were discarded. All procedures were carried out at 4°C or on ice.

### 4.8 Binding and uptake assays

The lysosomal binding and uptake assays were carried out as previously described (Nie et al., 2020). Briefly, isolated intact lysosomes were pre‐incubated with or without the protease inhibitor cocktail for 10 min and then incubated with lysates prepared from HEK293T cells overexpressing His‐Keap1 in MOPS buffer (10 mM 3‐[N‐morpholino] propanesulfonic acid [cat # M1254, Sigma], pH 7.3, 0.3 M sucrose) for 30 min at 37°C. After the incubation, all samples were centrifuged at 16,100 *g* (Eppendorf centrifuge, 5415R) for 5 min at 4°C, and the pellet fractions were washed 3 times with 100 μl of cold incubation buffer. The final pellet fractions were resuspended in loading buffer, subjected to SDS‐PAGE, and immunoblotted with anti‐His antibody. In the presence of inhibitors of lysosomal proteases, the protein detected represents the amount of protein binding with the lysosome membrane and taken up by lysosomes. And in the absence of inhibitors, the protein detected only reflect the part binding with the lysosome membrane.

### 4.9 In vitro ubiquitination assay

SN4741 cells were transduced with Flag‐LAMP2A or si‐*Lamp2a* plus HA‐Ub for 48 h. Then, cells were treated with 10 µM MG132 (cat # S2619, Selleck) for 5 h before assays. Cells were collected and lysed with RIPA buffer. The level of Nrf2 ubiquitination was determined by immunoprecipitation with an anti‐Nrf2 antibody followed by Western blot with an anti‐HA antibody.

### 4.10 ROS detection

Fluorescent dye 2′,7′‐dichlorofluorescin diacetate (H2DCF‐DA, cat # S0033, Beyotime) was used to measure the intracellular ROS. In brief, after transfection and 6‐OHDA treatment, cells were treated with 10 μM H2DCF‐DA for 30 min at 33℃, following by 2 times washing with prewarmed PBS. Then, cells were collected into FBS‐free DMEM, counted, diluted to the same concentration, and detected by microplate reader (SpectraMax M2^e^).

### 4.11 Quantitative real‐time PCR (qPCR)

Total RNA of cells was extracted and isolated using Trizol (cat # 11667165001, Roche). mRNA was reverse‐transcribed with random primers using First Strand cDNA Synthesis Kit (cat # 04896866001, Roche). Quantification of mRNA was performed using the iQ™5 PCR system (Bio‐Rad) with Hieff^®^ qPCR SYBR Green Master Mix (cat # 11201ES03, Yeasen) with 10 ng of total RNA in 20‐μl reaction mixtures, and thermal cycling conditions was set as follows: 1st step: 95℃ for 5 min with 1 cycle; 2nd step: 95℃ for 10 s and then 60℃ for 30 s with 40 cycles. Each sample was run in triplicate. Target mRNA was normalized to *β*‐*actin* mRNA. The normality tests were performed by D'Agostino and Pearson omnibus normality test using GraphPad Prism 6 software. The primer sequences used for real‐time PCR are listed in Table S1.

### 4.12 TUNEL staining

The terminal deoxynucleotidyl transferase dUTP nick end labeling (TUNEL) assay was performed with the In Situ Cell Death Detection Kit (cat # 11684817910, Roche) for apoptosis detection. SN4741 cells were seeded onto chamber slides, transfected with siRNA, and treated with 6‐OHDA for 24 h. Then, cells were fixed, permeabilized, and incubated with TUNEL reaction mixture at 37°C for 1 h as described in the manufacturer's protocol. After washed twice with PBS (pH 7.4), slides were stained with DAPI (cat # D9542, Sigma,) and observed under a laser scanning confocal microscope (NikonA1).

### 4.13 Immunofluorescence

SN4741 cells grown on chamber slides were fixed in 4% formaldehyde, permeabilized in 0.3% Triton X‐100, incubated with anti‐LAMP2A (cat # ab125068, Abcam) and anti‐Keap1 (cat # 60027‐1‐Ig, Proteintech) overnight at 4°C, and washed 3 times with PBS, then treated with Alexa Fluor 488‐adsorbed goat anti‐rabbit IgG (cat # A11008, Invitrogen) and Alexa Fluor 594‐adsorbed goat anti‐mouse IgG (cat # A11032, Invitrogen) for 2 h in the dark at room temperature. Nuclei were stained with DAPI 5 min to enable quantification of the total nuclear intensity. Cells were observed under a laser scanning confocal microscope (NikonA1) using a 60× oil immersion objective.

### 4.14 Statistical analysis

Statistical analyses were performed using GraphPad Prism 6 software, and the data are presented as mean ± standard error of mean (SEM) from at least three independent experiments. Statistical differences between group means were compared using Student's *t* test or two‐way ANOVA. A *p*‐value less than 0.05 was considered statistically significant.

## CONFLICT OF INTEREST

All authors declare no competing interests.

## AUTHOR CONTRIBUTIONS

L.Z. and Q.Y. conceived the study and designed the experiments. L.Z. and S‐L.H. performed the cell culture, Western blot, and qPCR assays. L.Z. and L.H. performed Co‐IP assays and TUNEL staining. T‐J.N., D‐N.R., and L.Z. performed lysosomal binding and uptake assays. K.T., L.X., and F‐F.L. participated the data analysis. Z‐X.M. provided advises about results analysis and paper writing. Q.Y. wrote the manuscript.

## Supporting information

Fig S1

Fig S2

Fig S3

Fig S4

Fig S5

Table S1

## Data Availability

The datasets used and analyzed during the current study are available from the corresponding author upon request.
