# Supplementary figures and images for: Chaperone‐mediated autophagy degrades Keap1 and promotes Nrf2‐mediated antioxidative response
Source: Aging Cell. 2022 May 10;21(6):e13616. doi: 10.1111/acel.13616 (PMC9197408; doi:10.1111/acel.13616)

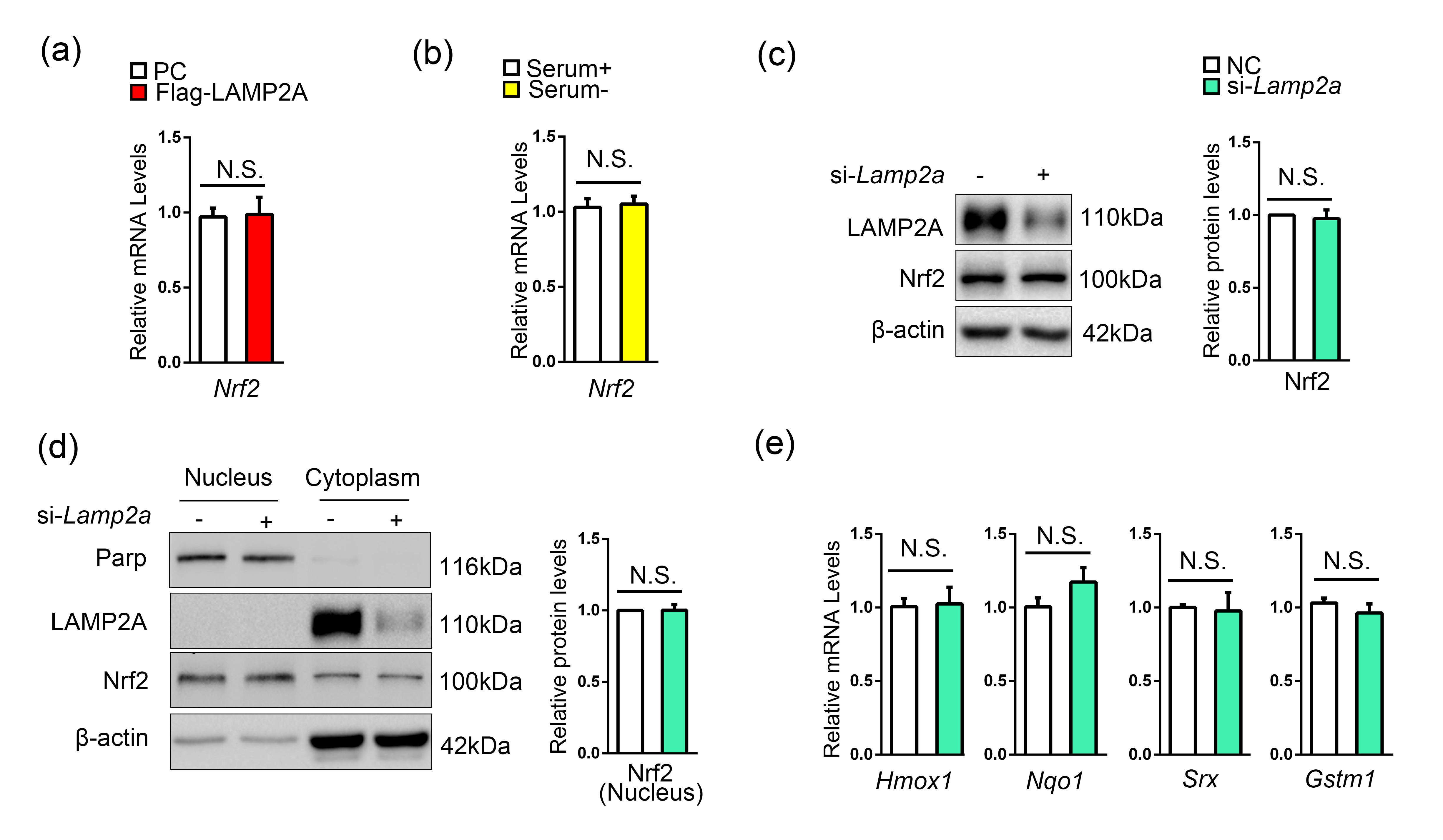

Supplement: Supplementary file 1 — Fig S1 [file ACEL-21-e13616-s001.tif]

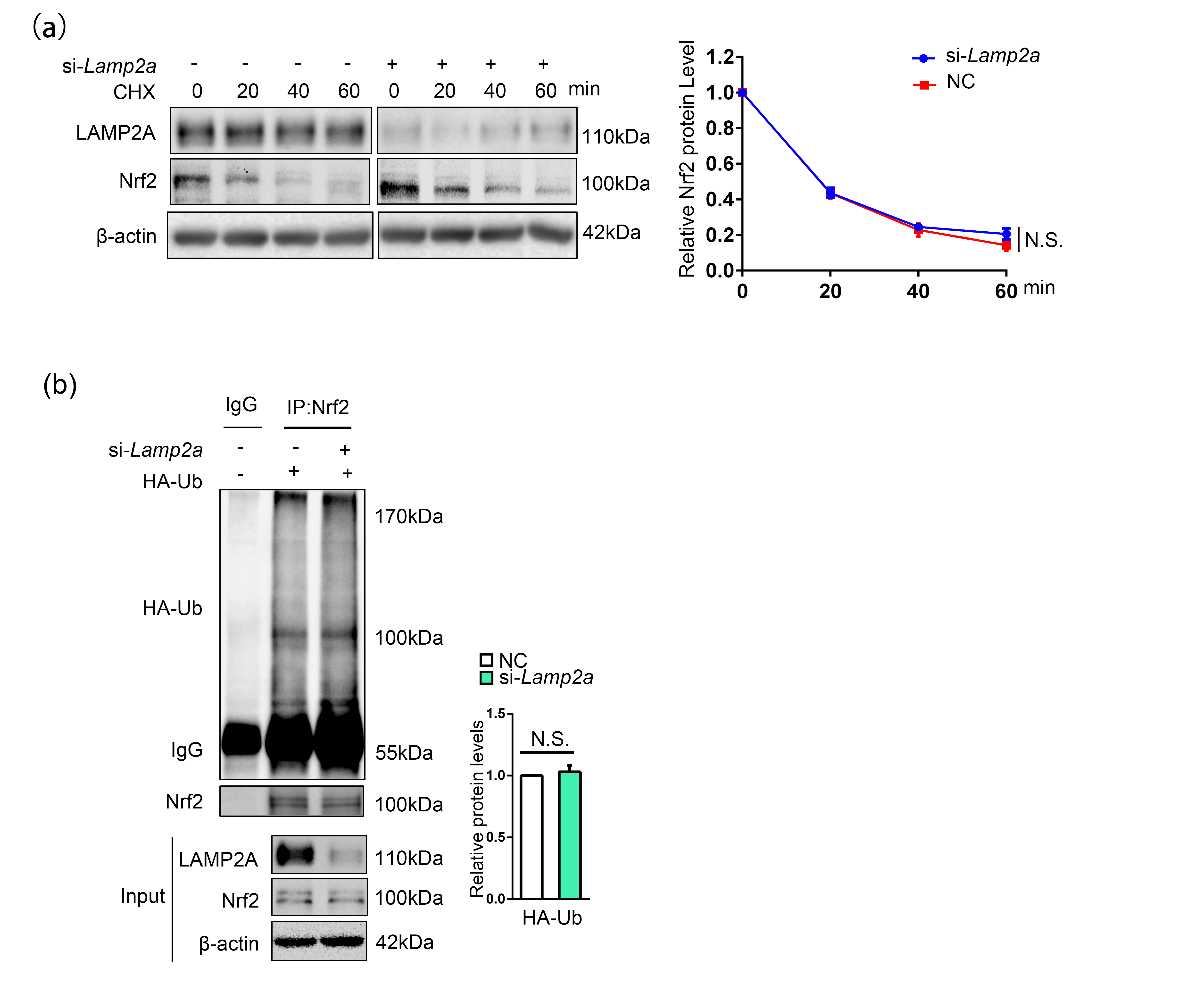

Supplement: Supplementary file 2 — Fig S2 [file ACEL-21-e13616-s004.tif]

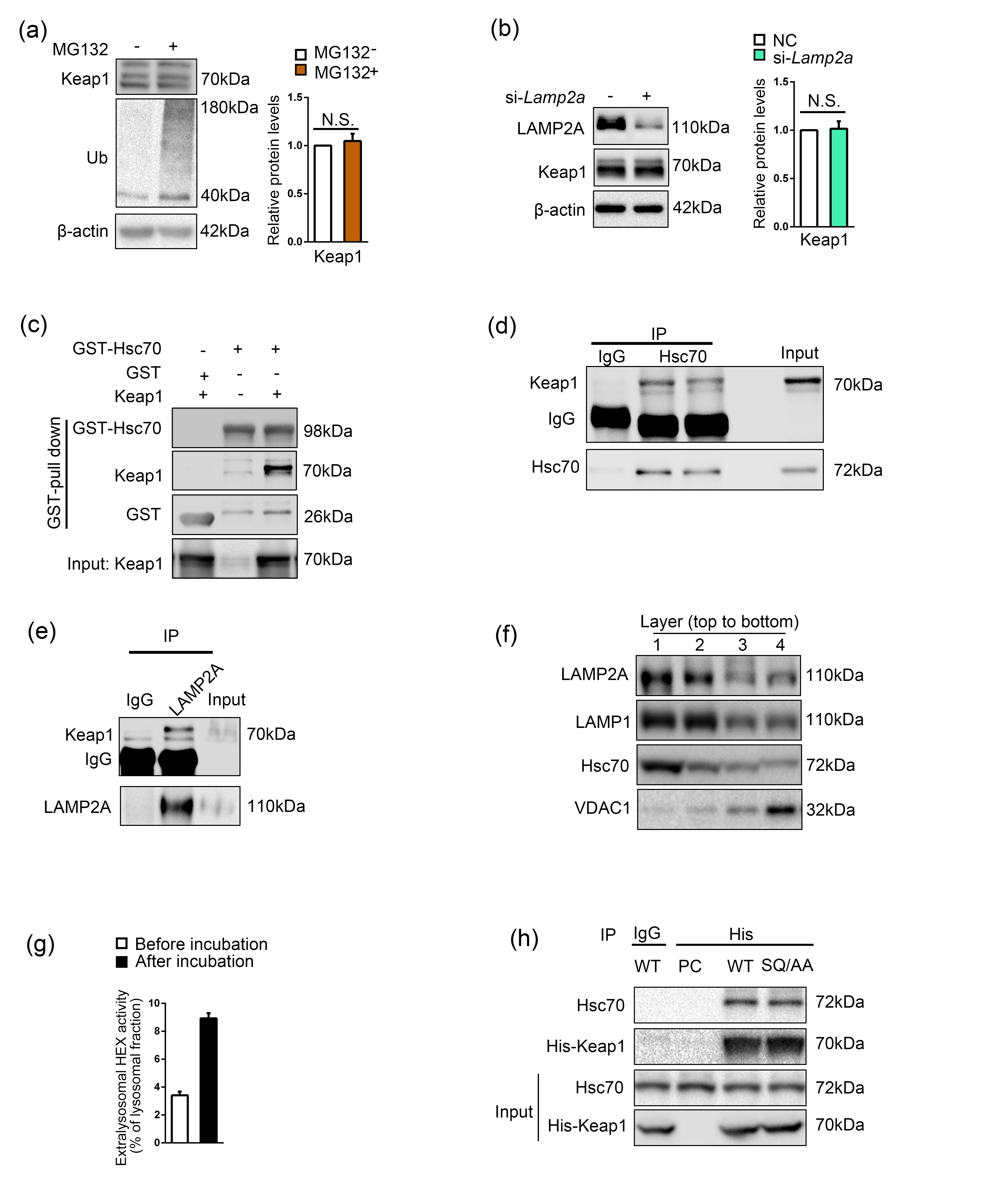

Supplement: Supplementary file 3 — Fig S3 [file ACEL-21-e13616-s006.tif]

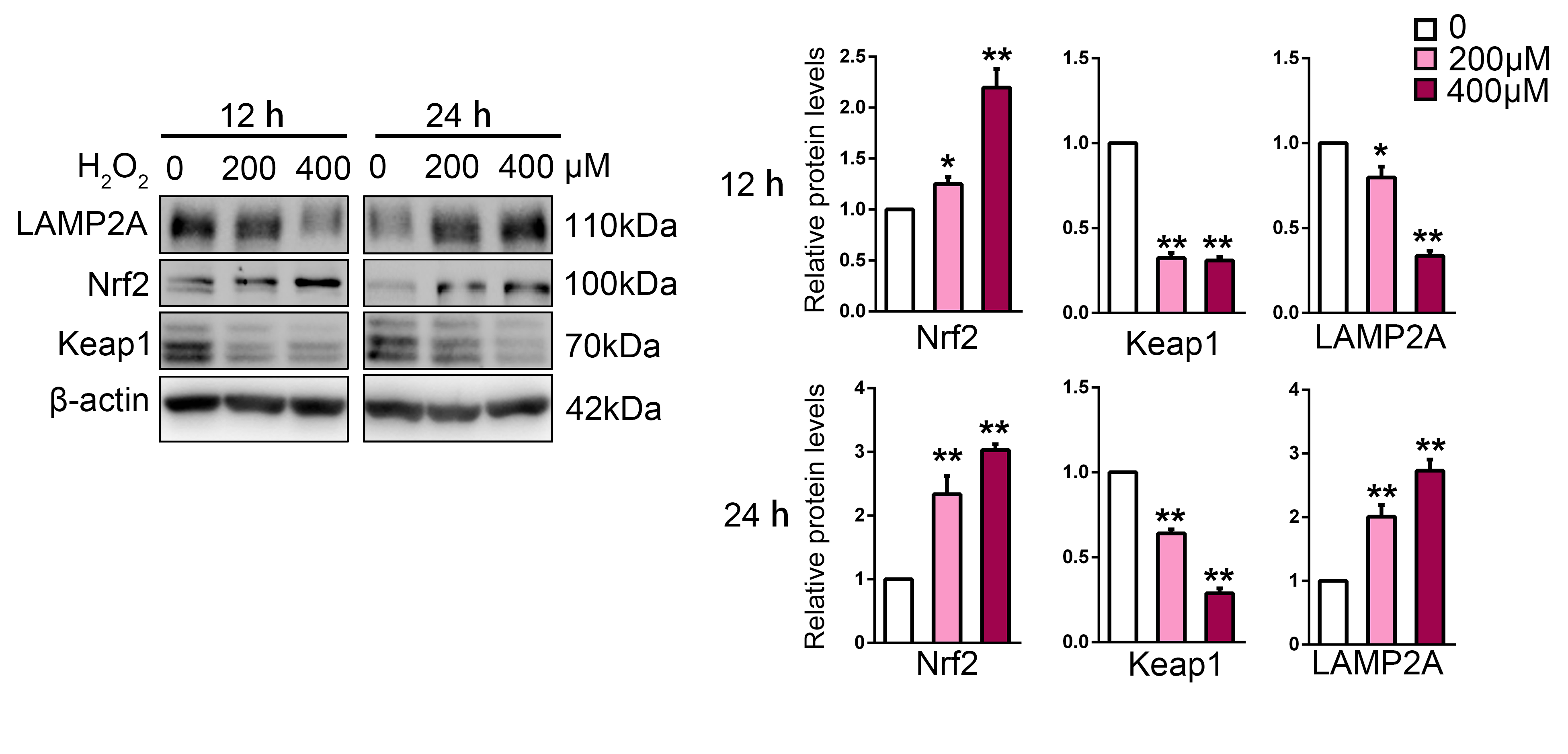

Supplement: Supplementary file 4 — Fig S4 [file ACEL-21-e13616-s003.tif]

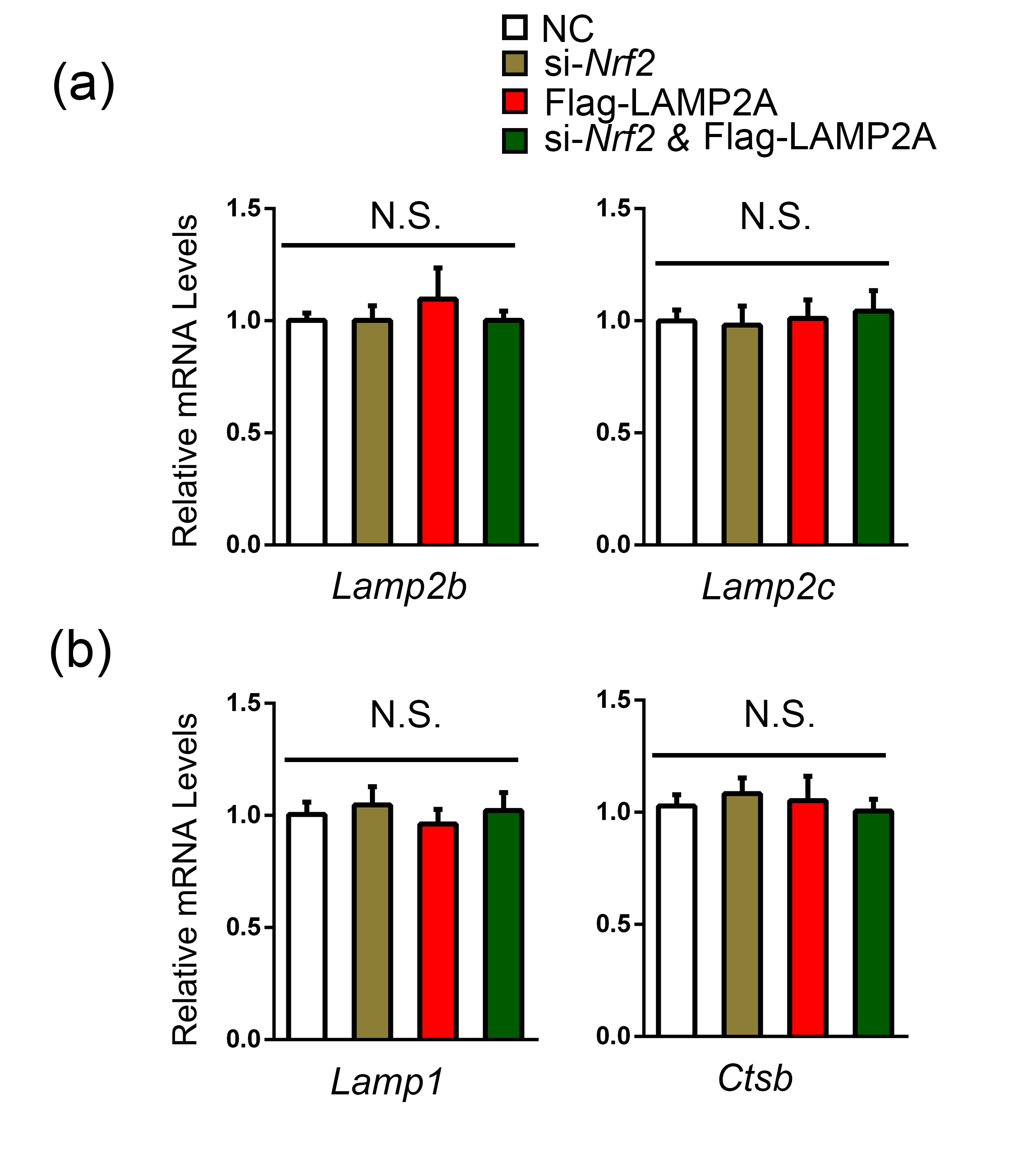

Supplement: Supplementary file 5 — Fig S5 [file ACEL-21-e13616-s005.tif]
