# Supplementary material for: Chaperone‐mediated autophagy degrades Keap1 and promotes Nrf2‐mediated antioxidative response
Source: Aging Cell. 2022 May 10;21(6):e13616. doi: 10.1111/acel.13616 (PMC9197408; doi:10.1111/acel.13616)
Supplement: Supplementary file 6 — Table S1 [file ACEL-21-e13616-s002.docx]

| Gene | Forward primer (5’-3’) | Reverse primer (5’-3’) |
| --- | --- | --- |
| *Homx1* | AAGCCGAGAATGCTGAGTTCA | GCCGTGTAGATATGGTACAAGGA |
| *Nqo1* | TTCTCTGGCCGATTCAGAG | GGCTGCTTGGAGCAAAATAG |
| *Srx* | GGAAGGAAGAAAGGAGATGGA | AGAGTTCAGGCTATGGGGATG |
| *Gstm1* | CTACCTTGCCCGAAAGCAC | ATGTCTGCACGGATCCTCTC |
| *Nrf2* | CTTTAGTCAGCGACAGAAGGAC | AGGCATCTTGTTTGGGAATGTG |
| *Lamp2a* | AGGTGCTTTCTGTGTCTAGAGCGT | AGAATAAGTACTCCTCCCAGAGCTGC |
| *Lamp2b* | ATGTGCTGCTGACTCGTGACCTCAA | TGGAAGCACGAGACTGGCTTGATT |
| *Lamp2c* | GGTGCTGGTCTTTCAGGCTTGATT | ACCACCCAATCTAAGAGCAGGACT |
| *Lamp1* | GACCCTGAAAGTGGAGAACAA | GGGCATCAGGAAGAGTCATATT |
| *β-actin* | ATATCGCTGCGCTGGTCGTC | AGGATGGCGTGAGGGAGAGC |
| *Ctsb* | TTAGCGCTCTCACTTCCACTACC | TGCTTGCTACCTTCCTCTGGTTA |
